# Re-analysis of publicly available methylomes using signal detection yields new information

**DOI:** 10.1038/s41598-023-30422-4

**Published:** 2023-02-27

**Authors:** Alenka Hafner, Sally Mackenzie

**Affiliations:** 1 Department of Biology, The Pennsylvania State University, 362 Frear N Bldg, University Park, PA 16802 USA; 2 Intercollege Graduate Degree Program in Plant Biology, The Pennsylvania State University, University Park, PA USA; 3 Department of Plant Science, The Pennsylvania State University, University Park, PA USA

**Keywords:** Computational biology and bioinformatics, Plant sciences, Epigenomics, DNA methylation

## Abstract

Cytosine methylation is an epigenetic mark that participates in regulation of gene expression and chromatin stability in plants. Advancements in whole genome sequencing technologies have enabled investigation of methylome dynamics under different conditions. However, the computational methods for analyzing bisulfite sequence data have not been unified. Contention remains in the correlation of differentially methylated positions with the investigated treatment and exclusion of noise, inherent to these stochastic datasets. The prevalent approaches apply Fisher’s exact test, logistic, or beta regression, followed by an arbitrary cut-off for differences in methylation levels. A different strategy, the MethylIT pipeline, utilizes signal detection to determine cut-off based on a fitted generalized gamma probability distribution of methylation divergence. Re-analysis of publicly available BS-seq data from two epigenetic studies in Arabidopsis and applying MethylIT revealed additional, previously unreported results. Methylome repatterning in response to phosphate starvation was confirmed to be tissue-specific and included phosphate assimilation genes in addition to sulfate metabolism genes not implicated in the original study. During seed germination plants undergo major methylome reprogramming and use of MethylIT allowed us to identify stage-specific gene networks. We surmise from these comparative studies that robust methylome experiments must account for data stochasticity to achieve meaningful functional analyses.

## Introduction

Cytosine methylation has been described as the fifth letter of the genetic code^1^, bestowing an additional level of information to the genome through regulation of gene expression^2,3^ and physical conformation of the DNA molecule^4^. However, the language of the methylome ^5^ has remained much more elusive to interpretation than that of the underlying nucleotide sequence. If we follow the DNA code analog, we must achieve proficiency in methylome decoding in at least three ways: (i) reading at single site resolution, (ii) interpretation of downstream effects of single position methylation status, and (iii) understanding the meaning of different methylation patterns in their local and global DNA context. While it is currently feasible to read cytosine methylation at single base resolution, owing to advances in whole genome sequencing through bisulfite conversion^6^, and to interpret high-density methylome changes, such as in transposable elements (TEs) during major developmental events or in methylation machinery mutants^2,3,7^, our proficiency in decoding of the methylome remains lacking.

Much of the early analysis of methylome variation focused on high-density methylation changes within defined intervals across the genome^8–10^. This methodology was likely a consequence of experimental emphasis on datasets deriving from DNA methylation machinery mutants, which produced extremely robust methylation signal^2,11^. These units of change were termed differentially methylated regions (DMRs) and varied across studies for window size, requisite differentially methylated position (DMP) number, and uniform directionality for hyper/hypo-methylation^12–14^. DMR analysis can identify genomic sites likely to undergo, or be released from, gene silencing^15^ and, therefore, often produce datasets rich in TE and heterochromatic genomic intervals. With these analytical approaches, however, the function of low density, intragenic methylation repatterning during development and in response to environmental stimuli remains unapproachable. While there is an extensive understanding of the evolutionary and mechanistic origin of gene body methylation (GbM)^16^, debate on its functionality remains^17^.

The plant methylome is often described as stochastic^18^. Variation is thought to arise from thermodynamic fluctuations of cellular machinery and the DNA molecule itself^5,19–22^. There is inherent stochasticity to methylome remodeling with each cell division, giving rise to ‘spontaneous epimutations’ in all methylation contexts^18,23,24^. Noise in BS-seq datasets is also amplified by tissue pooling as the plant epigenome appears to be developmental stage-, tissue- and cell-type specific^25–31^. Therefore, a full picture of environmental or developmental epigenetic responses is only possible by separating treatment signal from noise in the system. This type of approach would make interpretation of methylome data feasible at the organ or organism level, even as single cell BS-seq becomes feasible in plants^32^, as it is in animals^33^.

Stochastic methylome variation is often dismissed as information-free noise, inherent to the study of biological systems^34^. However, recent advances in computational biology demonstrate that interpreting existing methylome data at single read^35^ or single cytosine^32,36^ resolution yields new information. Single cytosine changes in methylome patterns have been shown to have important phenotypic effects^37,38^ and we are beginning to recognize the importance of gene body methylation in plants^16,39^. These observations point to a gap between the prevalent methodology and our current understanding of methylome biology.

Signal detection with machine learning is one approach to methylome data that bridges this gap. The MethylIT tool, based on physical statistics approaches, has been effective in identifying treatment-associated differential methylation in multiple study systems^5,36,40,41^. To test the relative efficacy of a signal detection-based method versus conventional methods for methylome data analysis, datasets produced in studies with robust experimental design and depth of sequencing can be re-analyzed with MethylIT, potentially increasing their utility. Here, we apply MethylIT, a signal detection pipeline, to two open access datasets of high quality; a study of methylome remodeling in response to phosphate starvation^42^ and an analysis of epigenetic changes during normal seed germination^29^. This report aims to demonstrate that applying novel approaches to existing high-quality methylome data can reveal meaningful additional information.

## Results and discussion

### Using signal detection approaches in methylome analysis avoids arbitrary filtering and accounts for stochasticity

Bisulfite deamination of DNA, by conversion of cytosine into uracil to be read as thymine, combined with whole-genome sequencing, has enabled the study of methylome variation in many organisms where an assembled reference genome is available. The cost of whole genome bisulfite sequencing (WGBS) continues to decline, enabling plant biologists to add epigenetic components to developmental and environmental response studies.

Upon completion of the bisulfite sequencing (BS-seq) runs, primary data must be converted to methylation counts for each cytosine in the genome. The first step, an overall quality check and the trimming of sequencing adaptors, is most commonly conducted with Trim Galore!, which performs both functions^43^. The resulting short sequence reads are then aligned to the reference genome based on the three different bases that result from bisulfite conversion. A prevalent aligner for this step is Bismark^44^, and the output file contains methylated and unmethylated counts for each cytosine in the genome as well as its context.

**Figure 1:**
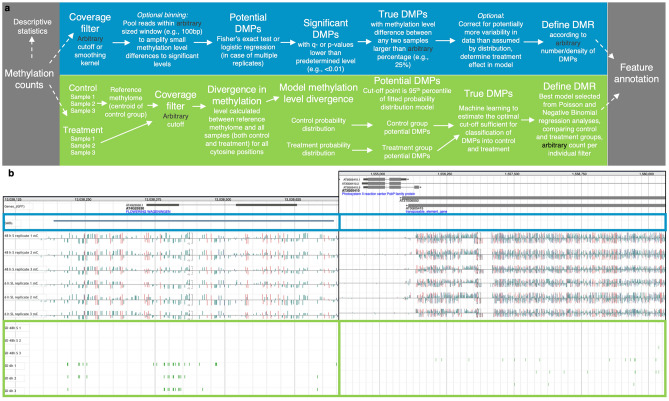
Comparison of MethylIT as a signal detection tool with representative common approaches. (**a**) Simplified representative pipelines of methylome analysis from methylation counts to differentially methylated regions (DMRs) via differentially methylated positions (DMPs). The blue panel is the generalized pipeline of the most prevalent approaches in the field, utilizing Fisher’s exact test or logistic regression, the green panel is the MethylIT pipeline, and the grey panels and dashed arrows represent steps in common. Arbitrary filtering steps and optional steps are highlighted in black and italics, respectively. (**b**) Methylation changes during two stages of seed germination, as detected by MethylIT and the hierarchical clustering approach employed in the original study by Narsai et al*.* (2017). The hierarchical clustering approach differs from the pipelines in Fig. 1a as it does not produce a DMP intermediate. The genome browser tracks from top to bottom are: annotated genes, DMRs found by hierarchical clustering^29^, 3 biological replicates of 48 h stratified seed and 3 biological replicates of seed after 6 h light exposure (raw methylation counts), 6 tracks of DMPs detected by MethylIT in each biological replicate. The blue rectangles highlight the common approach analysis results, and the green rectangles highlight the MethylIT results. Both the left and the right panels show a gene identified as a DMR with MethylIT, with the right panel gene not being deemed a DMR by hierarchical clustering. (Created in JBrowse^45^).

Selection of an analysis pipeline occurs at this stage, which can make a fundamental difference to final data output, both for differentially methylated positions (DMPs) and regions (DMRs). There are more than 20 bioinformatic tools available^14^ and they differ significantly in the statistical approaches implemented^25^. Without advanced statistical mathematics training, it is often easiest to adopt the most user-friendly, but not necessarily most powerful, options. Figure 1a compares MethylIT (a signal-detection pipeline) to the generalized pipeline of multiple other approaches. Figure 1b shows the methylation counts with DMRs (hierarchical clustering approach, blue panel) and treatment-associated DMPs (MethylIT, green panel) for two genes in a seed germination study^29^. This example demonstrates both the divergence of results when different methods are used and the bias of using a DMP-density-based approach (Fig. 1b, right).

The number of samples or biological replicates varies widely between methylome studies. The limiting factors include the cost per sample for WGBS and the amount of tissue needed (seedlings are often pooled into one sample). Whereas many common methylome analyses (Fig. 1a, blue panel) require only two samples (one control and one treatment), signal detection approaches require multiple (generally 3–5) biological replicates for each experimental condition to allow assessment of background noise (stemming from thermodynamic fluctuation) in the control group. It would not be valid to compare two samples with no replication using signal detection because treatment-associated DMPs would be indistinguishable from DMPs arising from stochastic variation. For this reason, control samples are pooled, and their centroid becomes the reference methylome, representing the background noise not associated with treatment.

The first step in identifying differentially methylated sites from all methylation counts is deciding which cytosines provide sufficient coverage to be included in further analysis. This step is largely arbitrary, and the importance of applying a noise-filtering solution here has been discussed before^13^. However, utilizing a smoothing kernel assumes that neighboring sites/regions exhibit correlated methylation levels, which holds only for CG and CHG but not CHH methylation contexts^13^. Both pipelines described in Fig. 1 use an arbitrary cut-off filter for coverage, followed by the identification of differentially methylated positions.

Some pipelines, including MethylSig, DMRcaller, and BSmooth^13,46,47^, implement tiling bins at this stage. This partitioning of the genome into arbitrary-sized regions pools reads to amplify small methylation level differences to significant levels. Most methods apply an appropriate statistical method after filtering for coverage, primarily Fisher’s exact test, beta-binomial or logistic regression when multiple replicates are considered^13,46–48^. This step yields a list of potential DMPs that are differentially methylated at the level of significance desired. “True” DMPs are a subgroup of potential DMPs that satisfy arbitrary criteria for differences in methylation levels between samples being compared. This cut-off varies widely between tools and users, ranging from 10 to 40%^12–14^, and often depends on the cytosine methylation context frequency distribution to minimize some of the bias. After a final list of DMPs is obtained, an optional step is a correction, in the event of greater variability in data than is assumed by the distribution, by determining the treatment effect in the statistical model. What follows is defining a DMR according to the number or density of DMPs present in a region. A region can be a genomic feature, a binned window size (e.g., 100 bp), or a sliding window. However, deciding on the number of DMPs sufficient for a DMR is a controversial step and another arbitrary filter.

MethylIT uses an information thermodynamics-derived approach to provide greater resolution of treatment-associated signals without consideration of DMP density ^5^. The first step distinguishing this pipeline calculates the difference in methylation levels between all given samples, including the controls and the reference (centroid of controls) methylome. In MethylIT, differences are estimated in the form of Hellinger divergence and total variation distance. Next, the divergences are modeled for all samples based on a generalized gamma probability distribution model, the fitted probability distribution, yielding separate control and treatment group probability distributions^5^. Others have also proposed using information theory approaches for analyzing methylome^32^. In MethylIT, the potential treatment-associated signal is above the 95th percentile of the fitted probability distribution of the methylation, which becomes the initial cut-off for each individual sample. The following step uses machine learning to distinguish true DMPs by estimating the optimal cut-off sufficient for the classification of DMPs into control and treatment (separately for all three methylation contexts, using several performance metrics). Conversely, MethylIT assesses each potential DMP based on the fitted probability distribution of methylation in each biological replicate separately, accounting for methylation heterogeneity and inherent stochasticity^40,41^. This type of filtering avoids the application of arbitrary cut-offs that can eliminate meaningful DMPs or include DMPs that are the result of stochastic fluctuation, both impacting outcomes.

Methylome analysis ends with feature annotation. The prevalent method yields a list of DMRs of arbitrary size that contain an arbitrary number of DMPs validated in the previous step. MethylIT also provides a list of DMRs; however, these are identified with the best generalized linear model selected from Poisson and Negative Binomial regression analyses, comparing control and treatment groups, and filtered with minimum methylation counts per cytosine and individual. The final step in both methodologies is to overlap or assess the proximity of DMR genomic addresses with genetic features of interest, including genes, TEs, and exons. Whereas prevalent methods look for overlaps of DMRs with annotated features, MethylIT statistically assesses all features of the selected category (i.e., genes, TEs, exons) as potential DMRs with no fixed set of DMRs to overlap with annotated features.

**Figure 2:**
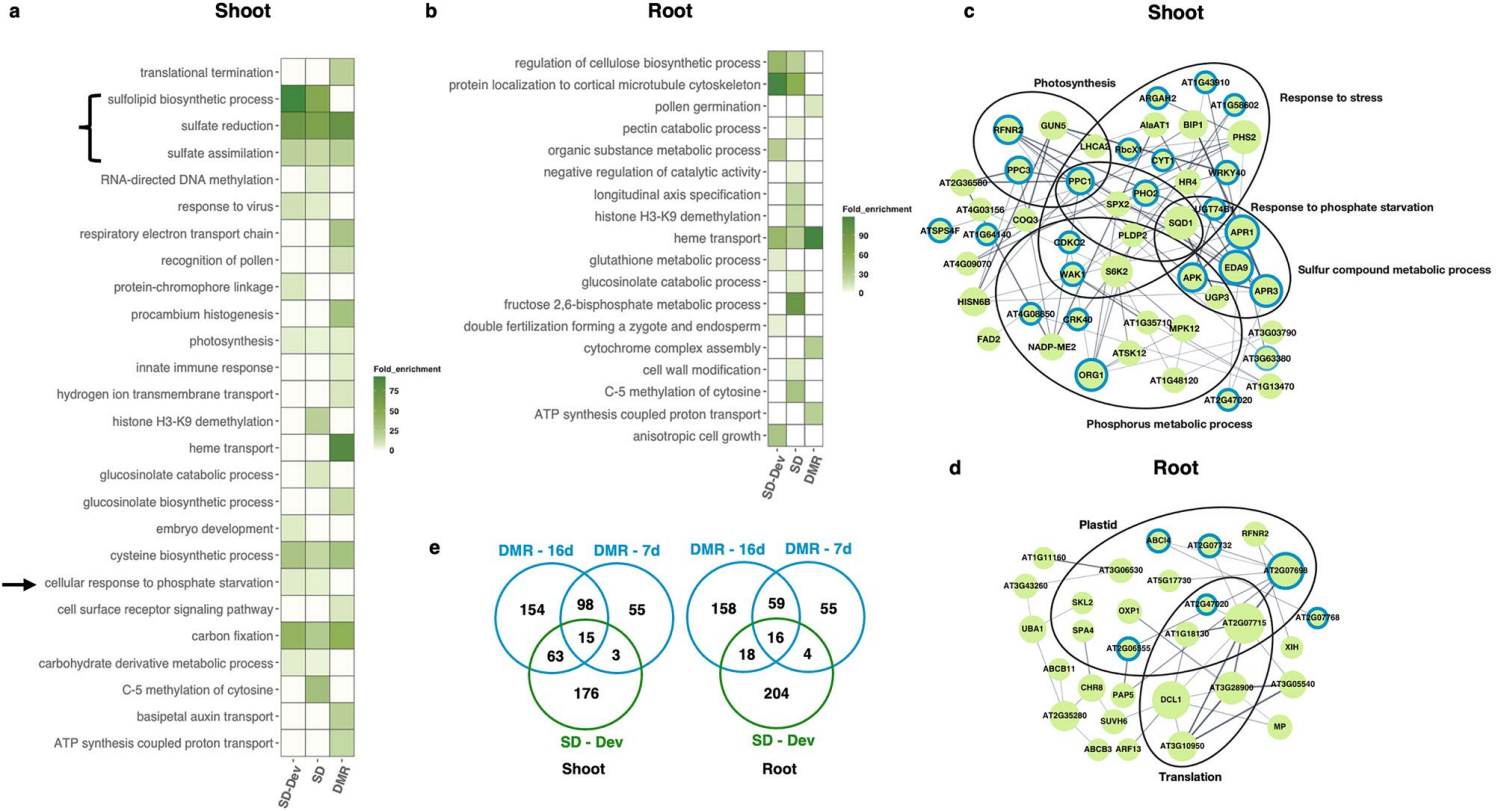
Phosphate starvation: methylome re-analysis using signal detection reveals additional gene networks of interest. BS-seq data obtained in the study^42^ was re-analyzed using MethylIT and compared to the original results. The result from the original study (DMR), signal detection with MethylIT (SD), and signal detection with subtracted development-associated DMGs (SD-Dev) for the 16-day starvation treatment are shown. (**a**) and (**b**) Heatmaps of tissue-specific GO terms with > fourfold enrichment obtained with DAVID^50^. Categories of interest are highlighted with a bracket and arrow. (**c**) and (**d**) Gene networks identified in Cytoscape^51^ from tissue-specific DMGs identified by MethylIT, with DMGs also identified in the original study circled in blue. The size of the cluster corresponds to the degree of connectivity score, nodes with less than 2 edges were removed and the genes are grouped according to Biological process GO terms. (**e**) Venn diagram of DMGs identified by both methods, MethylIT in green and the original study in blue. DMGs identified in the original study are shown for both 7 days and 16 days of phosphate starvation treatment.

**Figure 3:**
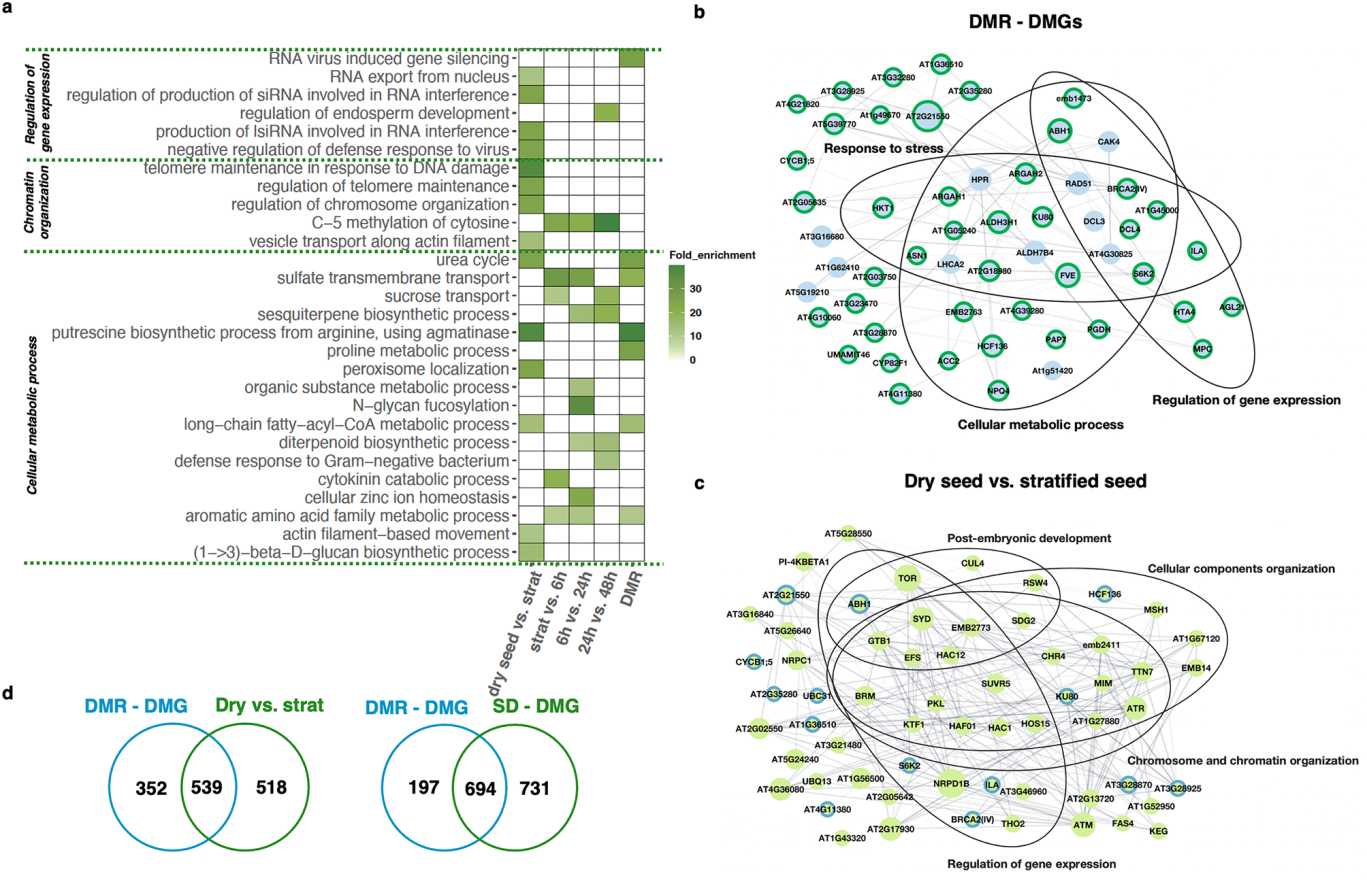
Re-analysis using signal detection reveals sequential methylome remodeling during seed germination. Bisulfite sequencing data obtained in the study^29^ was re-analyzed using MethylIT and compared to the original results. (**a**) Heatmap of Biological process GO terms with > tenfold enrichment (obtained with DAVID^50^), for MethylIT DMGs identified with pairwise analysis of individual stages during seed germination and genes overlapping with differentially methylated regions identified in the original study (DMR-DMGs). (**b**) and (**c**) Core gene networks identified in Cytoscape^51^. Genes are grouped according to Biological process GO terms. (**b**) Core hub network from DMGs identified in the original study, with the DMGs also identified by MethylIT circled in green. (**c**) Core hub network of DMGs identified by MethylIT in the first developmental transition, from dry seed to stratified seed; DMGs that were also identified in the original study are circled in blue. (**d**) Venn diagram of DMGs identified by both methods, MethylIT in green (first developmental transition on the left, all stages pooled on the right) and the original study in blue.

The resulting number of differentially methylated genes (DMGs) is generally higher using MethylIT than with the prevalent methodology (Table 1), which may raise concerns about insufficient filter stringency. However, the downstream functional analysis combined with MethylIT yields the same core gene networks with or without the most stringent filter cut-offs^14^. Figure 2e and 3d demonstrate the modest overlap between the original studies’ DMGs and those from MethylIT, which is the result of MethylIT (i) excluding DMRs caused by stochastic variation, (ii) adding DMRs with lower density DMPs that are nevertheless treatment-associated, (iii) only searching for DMGs and not other differentially methylated genomic regions for the sake of intelligibility (e.g., a gene can include differentially methylated exons but was not identified as a DMG). Additionally, we did not increase stringency simply to decrease the number of output DMGs as that can exclude biologically meaningful results (Fig. 1b).

**Table 1:** Summary of DMG numbers identified in the original studies of phosphate starvation^42^ and seed germination^29^ (DMR) and their overlap with DMGs identified using the MethylIT pipeline (SD).

Phosphate starvation				Seed germination			
	SD	Overlap	DMR		SD	Overlap	DMR
Root, 7d, + versus – Pi *	421	47	134	Dry versus stratified seed *	1057	539	891
Root, 16d, + versus – Pi *	609	139	251	Stratified seed versus 6 h light *	698	467	
Shoot, 7d, + versus – Pi *	518	54	171	6 h light versus 24 h light *	786	492	
Shoot, 16d, + versus – Pi *	695	188	330	24 h light versus 48 h light *	520	223	
Root, + Pi, 7d versus 16d *	582	^†^	^†^	Hub—Dry versus stratified seed ^‡^	62	13	56
Shoot, + Pi, 7d versus 16d *	623	^†^	^†^	Hub—Stratified seed versus 6 h light ^‡^	30	15	
Root, Pi starvation – development	242	139	251	Hub—6 h light versus 24 h light ^‡^	54	18	
Shoot, Pi starvation – development	257	78	330	Hub—24 h light versus 48 h light ^‡^	29	8	
Hub – Root ^‡^	30	1	12				
Hub – Shoot ^‡^	48	5	22				

* denotes which stages were compared when using MethylIT, with the first one listed acting as a reference and second as treatment. 7d and 16d refer to shorter and longer treatments, with + meaning control levels of phosphate and – the phosphate starvation. † means there was no comparable analysis available and ‡ denotes Cytoscape core hub (core k-means cluster with highest degree of connectivity, see Methods).

By comparing pipelines and their data outputs (Fig. 1), we can conclude that the different methods address fundamentally different questions about the methylome. The prevalent, “percentage of methylation”-gated methods endeavor to *describe global changes in methylome levels.* These changes generally include the percentage of methylated cytosines in each context, densely hypo- or hypermethylated or heterochromatic regions, TE silencing, and the global impact of epigenetic machinery mutants. MethylIT, as a signal detection method, aims to *read global changes in methylome patterns at single cytosine resolution.* The method, therefore, treats hypo- and hyper-methylation at each site equally, while evaluating each DMP based on the fitted probability distribution of each individual. The approach allows tracking of subtle changes in methylome repatterning without consideration of changes in methylome levels and discriminates variation within the treatment condition from the stochastic background effects present in both treatment and control conditions. Where most common approaches neglect to count DMPs that appear outside of “CpG islands”^49^, which have been shown to have important downstream and phenotypic effects^37,38^, the MethylIT procedure, by identifying only treatment-associated DMPs, incorporates all data regardless of methylation context or DMP density. Here, we focus our analysis on DMGs (not differentially methylated TEs or exons), particularly to demonstrate the importance of finding and interpreting intragenic methylation.

### Signal detection combined with functional annotation analysis reveals additional information

#### Phosphate starvation

A 2015 study of epigenetic responses to phosphate starvation in Arabidopsis revealed that methylome repatterning occurs in response to low phosphate (Pi), with altered expression of a small number of differentially methylated genes responsive to low phosphate conditions^42^. We selected this study for re-analysis with the MethylIT signal detection pipeline based on several features of its experimental design. The depth of sequencing was sufficient, each condition had three individual biological replicates with no pooling, controls were rigorous and present in both short- and long-term starvation conditions (7 days and 16 days), and data were collected for roots and shoots separately.

We conducted several pairwise comparisons between different experimental treatments with MethylIT. In all computations, the reference methylome was pooled from three control samples (Fig. 1a), which were always the shorter or no starvation condition. For shoot and root datasets separately, we compared methylomes for 7 days of low phosphate with 7 days high phosphate, 16 days of low phosphate with 16 days high phosphate, and 7 days high phosphate with 16 days high phosphate. The latter analysis was done to identify differentially methylated genes that result from normal development so that they could be later subtracted from the low phosphate treatment datasets. This analysis, using standard settings (see Supplementary Table S1), yielded a higher number of DMGs than the original DMR-based analysis (see Table 1 and Supplementary Table S2). Yong-Villalobos et al*.* (2015) identified DMPs using an F-test and subsequently DMRs as regions where DMP density exceeded global DMP density. Yong-Villalobos et al*.* point out the arbitrary nature of defining a DMR and hence conduct further analysis using both DMRs and all DMPs. For valid comparison with MethylIT, we use their reported list of DMRs overlapped with TAIR10 genes (see Supplementary Table S2). As a standard part of our analysis, DMGs were functionally analyzed with DAVID GO^50^ and Cytoscape^51^. To compare both methylome analysis pipelines, the DMGs identified in the original study were functionally analyzed alongside the MethylIT output.

Both analyses found tissue-specific methylome responses to phosphate starvation that were more pronounced after longer starvation treatment (Table 1). Figure 2a, b show that the pronounced biological processes identified among DMGs were similar yet distinct between the two pipelines. Cellular response to phosphate starvation, identified in both analyses, was only present as an enriched category using signal detection and was more pronounced when development-associated DMGs (differential methylation not associated with phosphate treatment) were subtracted. A subset of genes was unique to both analyses (Fig. 2e), which is the result of MethylIT only including treatment-associated DMGs and excluding stochastic variation. More DMGs overlapped with differentially expressed genes (DEGs) in the signal detection DMGs than DMR-DMGs from the original study (Supplementary Table S3). The GO term categories that were uniquely enriched in the DMR-based analysis largely contained DMGs that were also detected using MethylIT but were not the top categories in the MethylIT DMG context (Supplementary Table S4). For example, whereas heme transport was a pronounced category in the original study, its relative importance was diminished in the shoot with MethylIT, despite signal detection also finding these same heme-related genes. Both analyses yielded sulfate metabolism genes, which were also differentially expressed, but this category was not prominent in the original study dataset. A connection between sulfate homeostasis and phosphate starvation has been previously described^52,53^.

MethylIT-derived shoot DMG network produced in Cytoscape confirmed the phosphate, sulfate, carbon fixation and photosynthesis DMGs as vital to the response, together with other stress-responsive genes (Fig. 2a, c). Root DMGs indicated a distinctive growth response to phosphate starvation, with anisotropic cell growth, longitudinal axis specification, cellulose biosynthesis, and protein localization to cortical microtubules. This network analysis also pointed to altered gene expression and a potential plastid response (2b, d). Core hub genes produced in Cytoscape from DMR-based analysis were lesser in number and did not match categories enriched in GO term analysis to the same extent (Supplementary Fig. S1, S2).

This sample comparison demonstrates the power of using signal detection, in combination with several functional analyses, to uncover treatment-associated and biologically meaningful differential methylation patterns. MethylIT confirmed the presence of biological processes identified in the original study and added additional resolution with meaningful connections to other processes. Crucially, the robust biological and developmental controls (sufficient replicates, tissue-specificity, and comparison of high phosphate treated plants after 7 and 16 days) allowed the staggering number of DMGs to be converted into a Pi-starvation-associated subset of manageable size. Network analysis allowed potential key players in this tissue-specific stress response to be revealed.

#### Seed germination

In 2017, three studies published in *Genome Biology* investigated methylome remodeling during embryogenesis and germination^27–29^. Narsai et al. (2017) analyzed five developmental stages and reported progressive demethylation of the Arabidopsis genome during the seed-to-seedling transition. This transition coincided with changes in mRNA and siRNA populations. We selected this dataset for re-analysis with MethylIT based on its robust biological replication, depth of sequencing, and limited discussion of the functional identity of DMRs determined. BS-seq read files were provided for dry seed, seed after 48 h stratification, 6 h after light exposure, 24 h after light exposure, and 48 h after light exposure. The investigators conducted methylome analysis using HOME (v0.1) with default parameters for time series analysis, and the added cut-off for the difference in methylation levels was 20%. In contrast to the original study’s hierarchical clustering, MethylIT analysis was conducted pairwise, with consecutive stages of development serving as the reference (control) methylome for the identification of DMGs in each following stage. As the original study did not include DMG analysis, we overlapped their reported 12,654 DMRs with TAIR10 genes to obtain 891 DMGs (Supplementary Table S5). As in the phosphate starvation study, the seed germination data was likewise functionally analyzed with DAVID GO^50^ and Cytoscape^51^ alongside the MethylIT output.

Using standard MethylIT settings (Supplementary Table S1), our re-analysis yielded a comparable number of DMGs to Narsai et al. (2017) in each individual stage (Supplementary Table S5), with the largest number of DMGs present in the first developmental transition and an overall larger number of unique DMGs (Table 1). More DMGs overlapped with differentially expressed genes (DEGs) and genes showing isoform variation during seed germination when the signal detection pipeline was used (Supplementary Table S6). The re-analysis identified the dry-seed-to-stratified-seed transition as the major stage of methylome repatterning in seed germination, both in the number of DMGs and the number of unique biological processes revealed by GO term analysis (Table 1, Fig. 3a, d, Supplementary Table S7). Accompanying the increase in a subset of DEGs and miRNAs at this stage^29^, methylome changes were targeted to the regulation of gene expression and chromosome and chromatin remodeling (Fig. 3a). In the following three developmental transitions, we observed differential methylation in different GO-term categories, with germinating seed response to light exposure as prominent. Regulation of gene expression and chromatin organization declined in prominence as methylation targets, and methylation itself became an enriched pathway (Fig. 3a).

The identity of the key network hubs identified by Cytoscape also changed with each developmental stage (Fig. 3b, Supplementary Fig. S3-5). Only 22.6% of DMGs present in the first stage hub were also reported in the original study (Fig. 3c, circled in blue) with similar outcomes in other stages (Supplementary Fig. S3-5). In contrast, 80.4% of core hub DMR-DMGs in the original study were also identified with MethylIT (Fig. 3b, circled in green) Many of the key players (core hub genes) are also members of the DNA damage repair pathway, which is linked with chromatin remodeling in the seed^54^. In the DMR-DMGs, regulation of gene expression and cellular metabolic processes were present as categories of enriched Biological Process GO terms, however, the resolution of the signal-detection analysis was not present, demonstrated by the smaller number of unique GO terms.

When expression data is overlapped with DMR data (Supplementary Table S6), the enriched GO terms in each developmental transition are more informative than each dataset individually (Supplementary Table S7). For example, the GO category “photosynthesis” is enriched threefold in DMGs identified by MethylIT after the seed is first exposed to light (stratified seed vs. 6 h in light transition), not enriched in DMR-DMGs or RNA-seq data individually but is fivefold enriched in the overlap of MethylIT DMGs and RNA-seq genes in the stratified seed versus 6 h in light transition, and threefold enriched after the next stage of 24 h in light.

This re-analysis again highlights how signal detection can add novel insights to existing datasets and increase their utility. Epigenetic reprogramming in the early stages of seed germination appears to be targeted to gene regulation and chromatin organization genes, which was not evident in the original analysis. As the seed is exposed to light, numerous genes related to a variety of metabolic processes related to photosynthesis become differentially methylated, including those responsible for cytosine methylation itself.

### Considerations for meaningful methylome analysis

**Figure 4:**
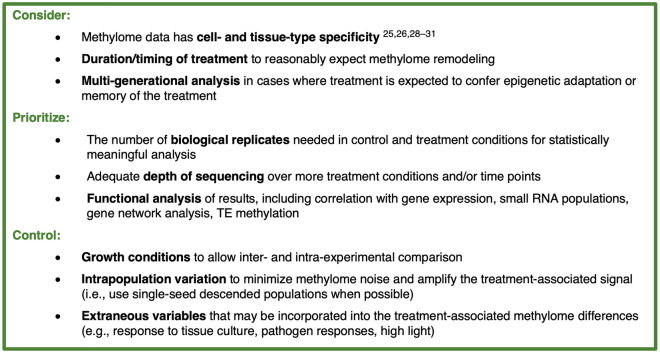
Suggestions and considerations on designing a methylome experiment that yields statistically meaningful results and data amenable to reuse.

Statistically meaningful methylome analyses require data coming from well-designed experiments; a poor dataset cannot be salvaged by stronger signal detection and/or machine learning algorithms. Figure 4 summarizes several considerations for an experimental scheme that will yield methylome datasets that can be used in signal detection pipelines and are likely to be amenable to reuse. Considering the often-prohibitive cost of processing robust numbers of biological replicates for BS-seq, we advise at least three biological replicates in the control group be prioritized over more time points or treatment conditions. This replication of controls is vital for allowing the calculation of background variation caused by methylome stochasticity in absence of the investigated treatment. With MethylIT as a signal detection pipeline, it is not appropriate to estimate a reference methylome without three control samples. Although the same number of replicates for the treatment group is advisable in order to confer confidence to treatment-associated DMPs, MethylIT can run the analysis with just one treatment group replicate.

Methylome analyses can yield hundreds or even thousands of differentially methylated genes and finding biological meaning in them can require multifaceted analysis. Searching the datasets for genes associated with the experimental condition investigated, i.e. an ad hoc approach, may uncover a novel epigenetic component of the system but does not, in itself, reveal new connections. However, when GO-term enrichment analysis is accompanied by gene network modeling, a genome-wide picture of the methylome response is constructed. The integration of k-means clustering of protein–protein interaction networks combined with gene ontology enrichment analysis has been shown to be a powerful approach to interpreting epigenetic data^36,55^. Figures 2a and 3a show GO-term enrichment obtained with the DAVID Functional Annotation tool^50^. Figures 2b, 3b, and 3c show the Cytoscape output for core gene network hubs that were obtained through unsupervised machine learning k-means clustering from DMGs identified with MethylIT and STRING protein–protein interactions data with Cytoscape. Even though categories of terms overlapped with those from GO-term enrichment analyses, the core gene hubs were not the most GO term-enriched categories, pointing to the importance of multifaceted analysis. These examples from phosphate starvation and seed development datasets demonstrate the need for multi-omics functional analysis that takes advantage of existing knowledge about biological connections for the true decoding of the methylome.

## Conclusions

We believe that signal detection, here applied in the form of the MethylIT, provides three key advantages over many prevalent methylome analysis approaches:

1. The ability to resolve data to interpretable networks
2. The ability to derive data with unambiguous outcomes (significance of enrichment or *p*-value discrimination) that provide confidence in conclusions with a minimum of “cherry picking”
3. The ability to derive meaningful new information that was simply not available using conventional methods to now conclude new pathway connections for phosphate starvation behavior and clearer stage discrimination for epigenetic and developmental transitions during seed development

The above-presented examples also reveal that it is the combination of gene expression and methylome data that is more informative than either dataset separately. Once signal detection-derived DMGs are overlapped with RNA-seq data, a smaller number of key biological processes and gene networks emerge. Crucially, they are at the intersection of the studied phenomenon (phosphate starvation and seed germination) and epigenetics, with C-5 methylation of cytosine always being one of the top scoring GO term enriched categories. MethylIT added numerous functionally important genes to the DMG list in both datasets, while removing stochastic variation-associated ones.

Because MethylIT does not rely on DMP density for the detection of DMRs, the bias of assuming only methylation-dense regions as biologically impactful (present in most methods) is eliminated. Instead, signal detection allows the identification of only treatment-associated DMPs, regardless of the proximity or direction of neighboring DMPs. This allows meaningful analysis of intragenic methylation repatterning, which remains the frontier of methylome analysis as it eludes density-dependent approaches. Our re-analysis also demonstrates the power of data reuse with computational approaches that were not available when data were generated, highlighting the importance of FAIR principles in increasing the utility of expensive datasets, like methylomes.

## Methods

### DMGs from original paper’s pipeline

To avoid any changes due to discrepancies in the reference genome annotation versions, we overlapped the DMRs reported in the original studies with version 38 of the TAIR10 genome annotation to obtain DMGs.

### DMGs from MethylIT (signal detection) pipeline

Raw sequencing reads were obtained from the GEO repository for both datasets. They were rimmed with TrimGalore and aligned the TAIR10 reference genome. DMPs were identified using MethylIT (version 0.3.2.4). Standard settings were used as described on the GitHub page (https://genomaths.github.io/methylit/articles/MethylIT.html), excluding modifications described in Supplemental Table S1. To identify the DMGs, we selected loci with at least three DMPs and minimum DMP density of 3 per 10 kbp, followed by group comparison using likelihood ratio test to select loci with log2fold change > 1 and adjusted *p*-value < 0.05. Gene annotation was done using version 38 of TAIR10.

### Functional DMG analysis

DAVID Functional Annotation tool (6.8)^50^ was used for GO term enrichment analysis of DMGs. GO terms with > fourfold (phosphate starvation) and > tenfold (seed germination) enrichment were plotted on heatmaps, using the ggplot2 package.

DMGs were also functionally analyzed using Cytoscape (3.9.1)^51^. The STRING database was used to construct the protein–protein interaction network from imported DMGs. The core hub was identified using the clusterMaker plug-in with the k-means cluster function. 3 clusters were calculated with 500 iterations, using Euclidean distance, betweenness centrality, closeness centrality, average shortest path length, clustering coefficient, degree, and eccentricity. The core hubs depicted in the figures were identified as the cluster with the highest centrality. The size of the node corresponds to the degree of connectivity score and the edge transparency corresponds to the STRING database score.

## Supplementary Information

Supplementary Information 1.

Supplementary Table S2.

Supplementary Table S3.

Supplementary Table S4.

Supplementary Table S5.

Supplementary Table S6.

Supplementary Table S7.

## Data Availability

The data that support the findings of this study are available in the supplementary material of this article. These data were derived from the following resources available in the public domain on the GEO repository: phosphate starvation data under accession GSE72770 (https://www.ncbi.nlm.nih.gov/geo/query/acc.cgi?acc=GSE72770) and seed germination data under accession GSE94459 (https://www.ncbi.nlm.nih.gov/geo/query/acc.cgi?acc=GSE94459).
