# Supplementary material for: Re-analysis of publicly available methylomes using signal detection yields new information
Source: Sci Rep. 2023 Feb 27;13:3307. doi: 10.1038/s41598-023-30422-4 (PMC9971211; doi:10.1038/s41598-023-30422-4)
Supplement: Supplementary file 1 — Supplementary Information 1. [file 41598_2023_30422_MOESM1_ESM.pdf]

Signal from noise: Methylome re-analysis using signal detection yields new information

Alenka Hafner[1,2] , Sally Mackenzie[1,3]*

[1] Department of Biology, The Pennsylvania State University, 362 Frear N Bldg, University Park, PA 16802, USA
[2] Intercollege Graduate Degree Program in Plant Biology, The Pennsylvania State University, University Park, PA, USA
[3] Department of Plant Science, The Pennsylvania State University, University Park, PA, USA
* Corresponding author

Supplementary Table S1. MethylIT R package settings, used for re-analysis of phosphate starvation and seed germination data

**Function**	**Settings (apart from default as on https://genomaths.github.io/methylit/articles/MethylIT.html)**
poolFromGRlist	stat = "sum"
estimateDivergence	Bayesian = TRUE,<br>min.coverage = c(16,4),<br>high.coverage = 450,<br>percentile = 0.999
getPotentialDIMP	dist.name = nlms_CHG$bestModel,<br>alpha = 0.05,<br>tv.col = 8,<br>tv.cut = 0.2
estimateCutPoint	clas.perf = TRUE,<br>classifier1 = "pca.qda"
countTest2	minCountPerIndv = 3,<br>countFilter = TRUE,<br>FilterLog2FC = TRUE,<br>test = "LRT",<br>CountPerBp = 0.0003,<br>Minlog2FC = 1,<br>pvalCutOff = 0.05,<br>MVrate = .95, maxGrpCV=c(1,1)

Supplementary Table S2. Differentially methylated genes identified using MethylIT and DMR-based analysis during phosphate starvation.

Supplementary Table S3. Overlap of differentially methylated genes identified using MethylIT and DMR-based analysis and genes differentially expressed under phosphate starvation.

Supplementary Table S4. Gene ontology term categories enriched in differentially methylated genes identified using MethylIT and DMR-based analysis in response to phosphate starvation. DAVID Knowledgebase (v2022q2) was used for GO function enrichment analysis.

Supplementary Table S5. Differentially methylated genes identified using MethylIT and DMR-based analysis during seed germination.

Supplementary Table S6. Overlap of differentially methylated genes identified using MethylIT and DMR-based analysis, differentially expressed genes and genes with isoform variation during seed germination.

Supplementary Table S7. Gene ontology term categories enriched in differentially methylated genes identified using MethylIT and DMR-based analysis during seed germination, and overlap with differentially expressed genes. DAVID Knowledgebase (v2022q2) was used for GO function enrichment analysis.

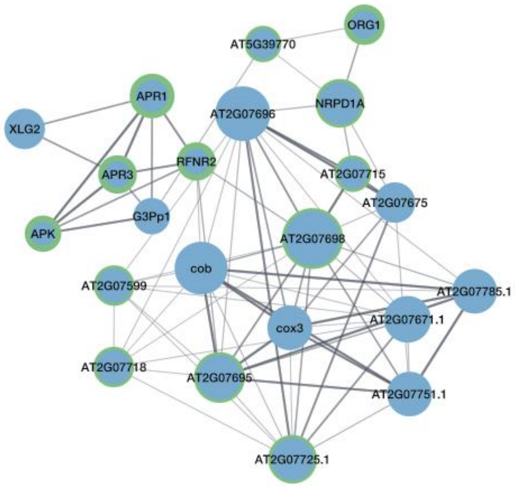

Supplementary Figure S1. Core differentially methylated gene (DMG) network hub in shoot tissue after 16 days of phosphate starvation modelled in Cytoscape. STRING networks from DMGs identified by Yong-Villalobos *et al.* (2015), with DMGs also identified by MethylIT circled in green. The core hub shown was identified from all DMGs using k-means clustering (Euclidean distance, 3 clusters, 500 iterations). Wider green outlines denote MethylIT DMG core hub genes. The size of the cluster corresponds to the degree of connectivity score.

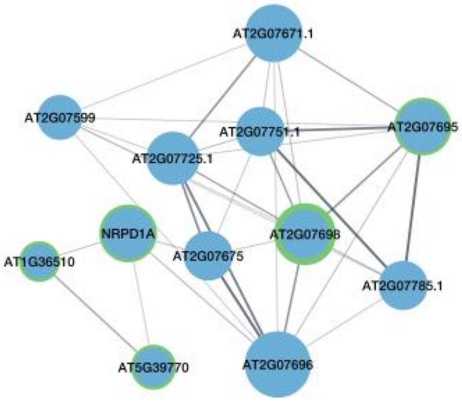

Supplementary Figure S2. Core differentially methylated gene (DMG) network hub in root tissue after 16 days of phosphate starvation modelled in Cytoscape. STRING networks from DMGs identified by Yong-Villalobos *et al.* (2015), with DMGs also identified by MethylIT circled in green. The core hub shown was identified from all DMGs using k-means clustering (Euclidean distance, 3 clusters, 500 iterations). Wider green outlines denote MethylIT DMG core hub genes. The size of the cluster corresponds to the degree of connectivity score.

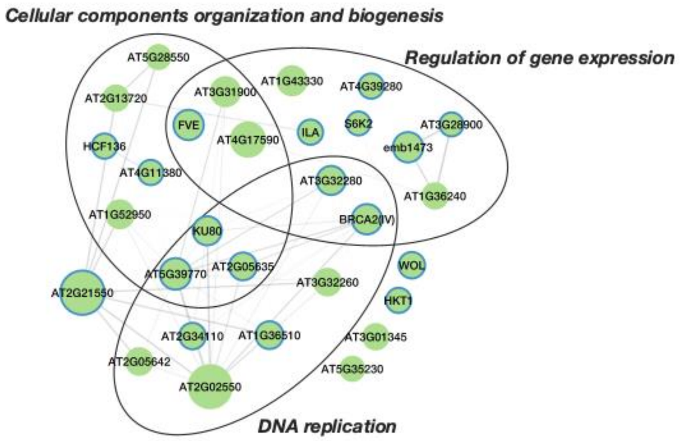

Supplementary Figure S3. Core hub network of DMGs identified by MethylIT in the second developmental transition, from stratified seed to 6h in light; DMGs that were also identified in the original study are circled in blue. The size of the cluster corresponds to the degree of connectivity score, edges with scores <0.5 are not shown. The core hub shown was identified from all DMGs using k-means clustering (Euclidean distance, 3 clusters, 500 iterations) and the genes are grouped according to Biological process GO terms.

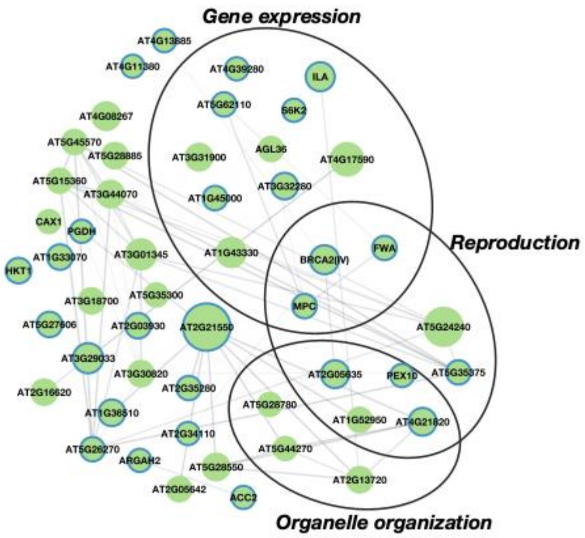

Supplementary Figure S4. Core hub network of DMGs identified by MethylIT in the third developmental transition, from 6h to 24h in light; DMGs that were also identified in the original study are circled in blue. The size of the cluster corresponds to the degree of connectivity score, edges with scores <0.5 are not shown. The core hub shown was identified from all DMGs using k-means clustering (Euclidean distance, 3 clusters, 500 iterations) and the genes are grouped according to Biological process GO terms.

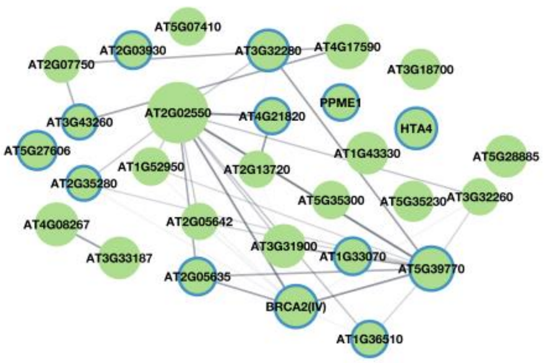

Supplementary Figure S5. Core hub network of DMGs identified by MethylIT in the fourth developmental transition, from 24h to 48h in light; DMGs that were also identified in the original study are circled in blue. The size of the cluster corresponds to the degree of connectivity score, edges with scores <0.5 are not shown. The core hub shown was identified from all DMGs using k-means clustering (Euclidean distance, 3 clusters, 500 iterations).
